# miR-488-5p mitigates hepatic stellate cell activation and hepatic fibrosis via suppressing TET3 expression

**DOI:** 10.1007/s12072-022-10404-w

**Published:** 2022-08-24

**Authors:** Jiannan Qiu, Shasha Wu, Peng Wang, Yan Zhou, Zhongxia Wang, Yong Sun, Chunping Jiang

**Affiliations:** 1 Department of Hepatobiliary Surgery, The Affiliated Drum Tower Hospital of Nanjing University Medical School, 321 Zhongshan Road, Nanjing, 210000 Jiangsu People’s Republic of China; 2 Department of Clinical Medicine and Rehabilitation, Jiangsu College of Nursing, Huai’an, 223005 People’s Republic of China; 3 Department of Hepatobiliary Surgery, The Affiliated Huaian No. 1 People’s Hospital of Nanjing Medical University, Huai’an, 223001 Jiangsu People’s Republic of China; 4 Hepatobiliary Center, The First Affiliated Hospital of Nanjing Medical University, Nanjing, 210029 Jiangsu People’s Republic of China; 5 Department of Hepatobiliary Surgery, Drum Tower Clinical College of Nanjing Medical University, Nanjing, 210000 Jiangsu People’s Republic of China

**Keywords:** Hepatic fibrosis, miR-488-5p, Hepatic stellate cells, TET3, TGF-β signaling pathway, CCL_4_, HFD, BDL, Extracellular matrix deposition, Target gene

## Abstract

**Background and aims:**

Numerous studies have demonstrated that hepatic fibrosis, a progressive condition as an endpoint of multiple chronic hepatic diseases, is largely characterized with the extensive activation of hepatic stellate cells (HSCs). The precise effect of miR-488-5p in HSCs during hepatic fibrosis has not been elucidated.

**Methods:**

In our study, qRT‐PCR was applied to assess the level of miR-488-5p in activated HSCs stimulated by TGF-β1. We built murine liver fibrosis models with carbon tetrachloride (CCl_4_), high-fat diet (HFD) and bile duct ligation (BDL). In vitro, the effects of miR-488-5p in HSCs were examined through cell proliferation assay and apoptosis. Luciferase reporter assay was applied to identify the underlying target of miR-488-5p. In vivo, the effects of miR-488-5p were explored through mouse liver fibrosis models.

**Results:**

The reduction of miR-488-5p in the activated HSCs induced by TGF-β1 and three mouse hepatic fibrosis models were identified. The in vitro functional experimentations verified that miR-488-5p restrained expression of fibrosis-related markers and proliferative capacity in HSCs. Mechanically, we identified that miR-488-5p inhibited tet methylcytosine dioxygenase 3 (TET3) expression via straightly binding onto the 3′ UTR of its mRNA, which sequentially restrained the TGF-β/Smad2/3 pathway. TET3 inhibition induced by the overexpression of miR-488-5p reduced extracellular matrix deposition, which contributed to mitigating mouse liver fibrosis.

**Conclusion:**

We highlight that miR-488-5p restrains the activation of HSCs and hepatic fibrosis via targeting TET3 which is involved in the TGF-β/Smad2/3 signaling pathway. Collectively, miR-488-5p is identified as a potential therapeutic target for hepatic fibrosis.

**Supplementary Information:**

The online version contains supplementary material available at 10.1007/s12072-022-10404-w.

## Introduction

Hepatic fibrosis, as a self-repair mechanism, is quite common when stimuli such as alcohol, hepatitis B virus (HBV), carbon tetrachloride (CCl_4_), and so on remain in the liver for the early stage. However, under the effect of the longtime harmful factors, liver fibrosis will sequentially transform into liver cirrhosis, hypohepatia and even hepatocellular carcinoma [1, 2]. The massive extracellular matrix (ECM) deposition and matrix remodeling are main characteristics of hepatic fibrosis which is involved with the activation of hepatic stellate cells (HSCs) [3–5]. The activated HSCs, stimulated by plentiful factors containing transforming growth factor β (TGF-β), are considered as the vital fibrotic elements [6–8].

MicroRNAs (miRNAs), belonging to a class of small non‐coding RNAs, serve as post-transcriptional regulators by negatively regulating downstream target gene expression [9, 10]. Increasing researches have documented the function of miRNAs in HSCs activation, which plays an essential role in the development of hepatic fibrosis. As previously reported, miR-130b-5p enhances the activation of HSCs and the severity of hepatic fibrosis via restraining SIRT4 expression, which is related with AMPK/TGF-β signaling pathway [11]. miR-98-5p suppressed HBV-related hepatic fibrosis via targeting TGFβ receptor 1B [12]. It is reported that miR-96-5p reduces HSCs activation through impeding autophagy via inhibiting ATG7 expression [13]. Even so, the effect of miRNAs in hepatic fibrosis remains indistinct.

The purpose of our research was intended to detect the role of miR-488-5p in hepatic fibrosis. First, the data presented that miR-488-5p were remarkably downregulated in the activated HSCs stimulated by TGF-β1 and mouse liver fibrosis models. Degree of activation of HSCs was restrained by miR-488-5p overexpression, as evidenced by the inhibited proliferation, promoted apoptosis and the reduction of profibrotic marker expression. We identified that tet methylcytosine dioxygenase 3 (TET3), serving as a target gene of miR-488-5p, played a pivotal role in hepatic fibrosis. More importantly, the degree of hepatic fibrosis attributed to CCl_4_, high-fat diet (HFD) and bile duct ligation (BDL) would be mitigatory once the mice were treated with ago-miR-488-5p. To sum up, our research findings demonstrate that miR-488-5p acts as a vital role in the hepatic fibrosis and might be a possible therapeutic target.

## Materials and methods

### Patient samples and cell line

All fibrotic specimens from patients with advanced liver disease and normal tissues from patients with hepatic hemangioma were collected in the first Affiliated Hospital of Nanjing Medical University. All samples were straightly frozen in liquid nitrogen once the surgical resection was completed.

We purchased the LX-2 cells, as a kind of cell line of hepatic stellate cells (HSCs), from the Cell Center of Shanghai Institutes for Biological Science. LX-2 cells were cultured with the Dulbecco’s Modified Eagle Medium (DMEM; Gibco, USA) which contained 10% fetal bovine serum (FBS) and 1% antibiotics (streptomycin/penicillin; Gibco, USA). Addition of TGF-β1 (10 ng/mL) into DMEM with no serum was intended to activate the LX-2 cells for 0, 3, 6, 12, and 24 h.

### Murine hepatic fibrosis models

Male C57BL/6 mice, 8 weeks old, were obtained and fed based on standard conditions at the animal house of Nanjing Medical University. Treatment with CCl_4_ (10% in olive oil, 2 mL/kg) through intraperitoneally injection twice a week for 8 weeks was intended to establish the hepatic fibrosis model. Meanwhile, olive oil (2 mL/kg) was used by the same way as the control group. The high-fat diet (D12492; Research Diets) was used to feed the mice for 24 weeks to establish the HFD model. Meanwhile, normal diet was used in the control group. For the control and bile duct ligation (BDL) group, sham or BDL surgery was performed in the mice, respectively.

### Western blotting

The RIPA buffer embracing 1% protease inhibitor and 1% PMSF was employed to extract the total protein based the standard protocol from the manufacturer (Beyotime). The extracted samples were separated via 10% SDS-PAGE and sequentially transferred to PVDF membranes. All antibodies were used in this study: TET3, TIMP-1 (Abcam), TGF-β, collagen-I, α-SMA, Smad2, p-Smad2, p-Smad3, Smad3, and GAPDH rabbit antibodies and HRP-conjugated anti-rabbit IgG antibodies (Cell Signaling Technology).

### qRT-PCR

The TRIzol reagent was employed to extract total RNA on the basis of the standard instructions. Subsequently, RNA reversion and cDNA synthesis were carried out through using Reverse Transcription Kit (Vazyme). mRNA amplification and cDNA quantification were carried out through using SYBR green (Vazyme) on the basis of standard protocols. The levels of miR-488-5p and U6 were assessed via using TaqMan miRNA assay system (Life Technologies Corporation). The normalization of genetic level rests with U6 or β-actin level, respectively. The primers used are presented in Supplementary material.

### Transfection

miR-488-5p mimics, scrambled miRNA (SCR-miRNA), TET3 lentivirus (LV-TET3), and control lentivirus (LV-NC), ago-miR-488-5p and ago-miR control were obtained from GenePharma. Transfection process was auxiliary with the existence of lipofectamine 2000 (Invitrogen) for 48 h for further researches. Mice were subjected to the treatment with ago-miR control and ago-miR-488-5p (20 nmol/200 mL) through tail injection. Once establishing the CCl_4_ and HFD liver fibrosis models for 2 weeks was finished, we administrated ago-miR control and ago-miR-488-5p into the mice twice a week. After the treatment with ago-miR control and ago-miR-488-5p twice a week for 2 weeks, the liver tissues from BDL model mice were obtained. Eventually, we collected the liver tissues for further detection.

### Colony formation and cell counting kit-8 assay

We planted LX-2 cells into 6-well plates at a density of 1000 cells per well and cultured for 15 days. The supernatant was discarded, and the clone was fixed with 4% paraformaldehyde. Staining was performed using a solution of crystal violet (staining time: 30 min). Eventually, the number of clone formations was counted.

We planted LX-2 cells into 96-well plates at a density of 2000 cells per well and cultured them with DMEM supplemented with CCK-8 reagent (10 μL/well) for 5 days to evaluate the cell viability. Once incubation for 2 h was completed, the absorbance (450 nm) of each well was observed.

### Apoptosis assay

The apoptotic level of LX-2 cells was evaluated through the Apoptosis Detection Kit (Vazyme Biotech Co., Ltd). After incubation with 5 μL Annexin V-FITC and 5 μL PI for 20 min, each cell sample was mixed with 400 μL binding buffer. All steps were followed by manufacturer’s protocol.

### Liver histopathology and fibrosis measurement

After liver tissues were totally fixed with 4% paraformaldehyde, they were embedded in paraffin. Subsequently, the paraffin block was made into slice and dyed with hematoxylin and eosin (HE). The Masson’s-stained or Sirius red’s-stained sections were used to assess the degree of liver fibrosis using the METAVIR scoring method. Eventually, the positive area was evaluated by pathologist to present the collagen deposition.

### Immunohistochemical (IHC) staining

After we obtained the tissue samples, the paraffin embedding was completed followed by standard steps. The paraffin block was made into slices (4 μm) which were incubated with anti-TET3 and anti-α-SMA for 12 h at 4 °C. Afterwards, the secondary antibody conjugated with HRP was utilized to incubate the slices for 1 h and eventually were dyed with 3,3′-diaminobenzidine and hematoxylin.

### Immunofluorescence (IF) staining

All steps obeyed to the standard protocols of IF staining. In short, LX-2 cells were incubated with primary antibody α-SMA, and followed by secondary goat anti-rabbit Texas Red-conjugated IgG. At the same time, DAPI was used to present the nuclei.

### Luciferase reporter assay

The luciferase reporter vector embracing the wild-type sequences of 3′‐UTR of TET3, and the mutated vector was constructed based on the binding sites. The luciferase reporter vectors were then co-transfected with miR‐488‐5p mimic or miR-SCR. At 48 h after transfection, cells were harvested and tested with the Dual-Luciferase reporter assay system (Beyotime, Shanghai, China) followed with manufacturer’s protocol. The relative luciferase activity was assessed as the ratio between firefly and Renilla luciferase activities detected by a dual-luciferase system (Beyotime, Shanghai, China).

### Statistical analysis

Statistical analysis results were shown as mean ± SEM. The SPSS software and GraphPad Prism were employed to manage the data and calculate the *p* value. Once *p* value < 0.05, was considered statistically significant.

## Results

### Downregulation of miR-488-5p is detected in activated HSCs

**Fig. 1:**
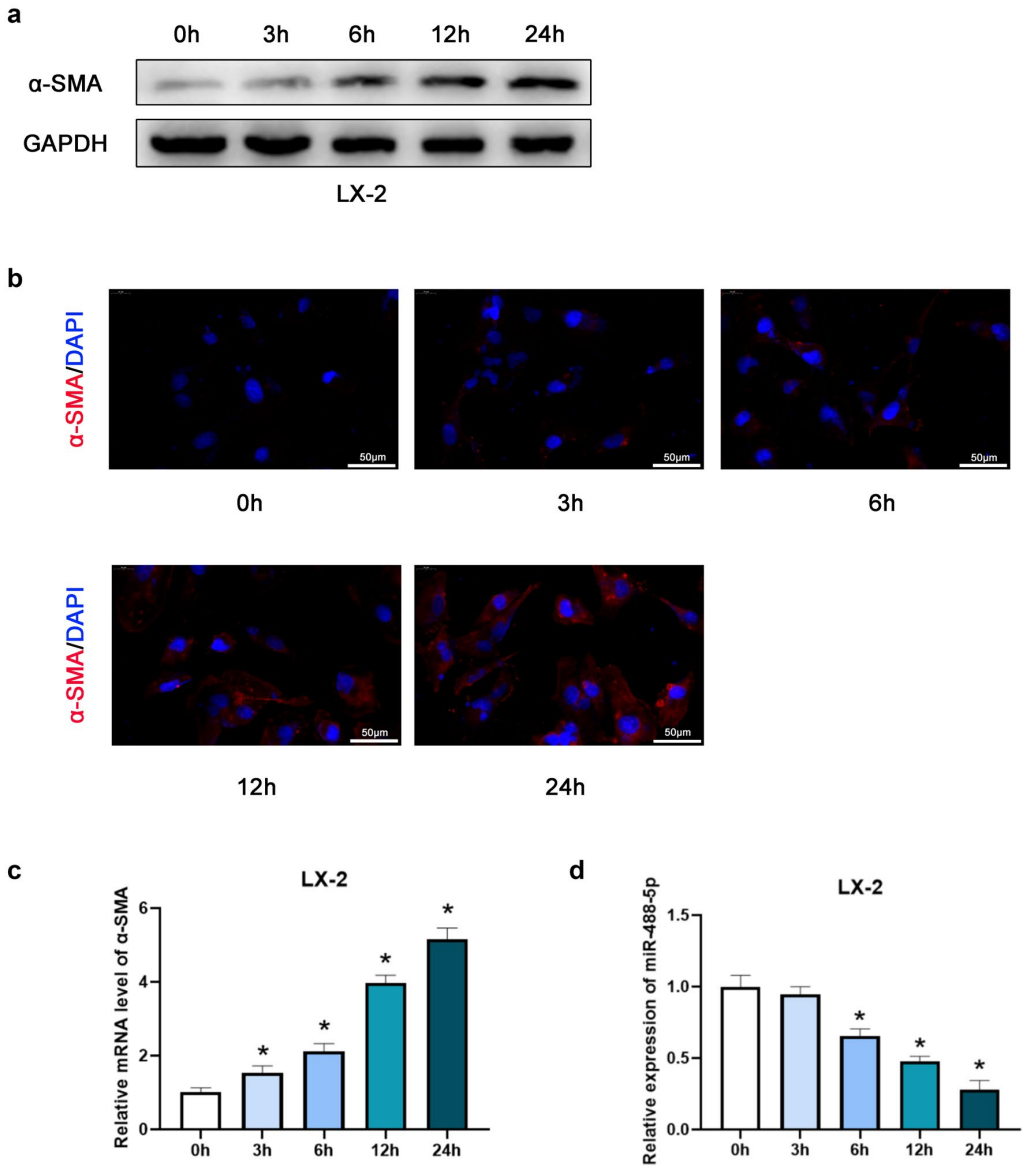
miR-488-5p is downregulated during HSCs activation. LX-2 cells were activated by TGF-β1 (10 ng/mL) for respective time. **a**, **b** Western blot (WB) and Immunofluorescence (IF) analysis of the expression level of α-SMA in LX-2 cells. **c**, **d** qRT-PCR analysis of the level of α-SMA and miR-488-5p in activated LX-2 cells. Data represent means ± SEM of at least three independent experiments. *p value < 0.05

First, the LX-2 cells were cultured with medium containing TGF-β1 (10 ng/mL) for 0, 3, 6, 12 and 24 h. As shown in the Fig. 1a, b, the expression of α-SMA was upregulated by degrees based on the results of WB and IF staining. Afterwards, we performed the qRT-PCR assay, the data revealed that as the relative level of α-SMA increased in the activated HSCs, the relative level of miR-488-5p was gradually downregulated (Fig. 1c, d). To sum up, our study presented that miR-488-5p is downregulated in the activated HSCs.

### Downregulation of miR-488-5p is detected in multiple hepatic fibrosis models

**Fig. 2:**
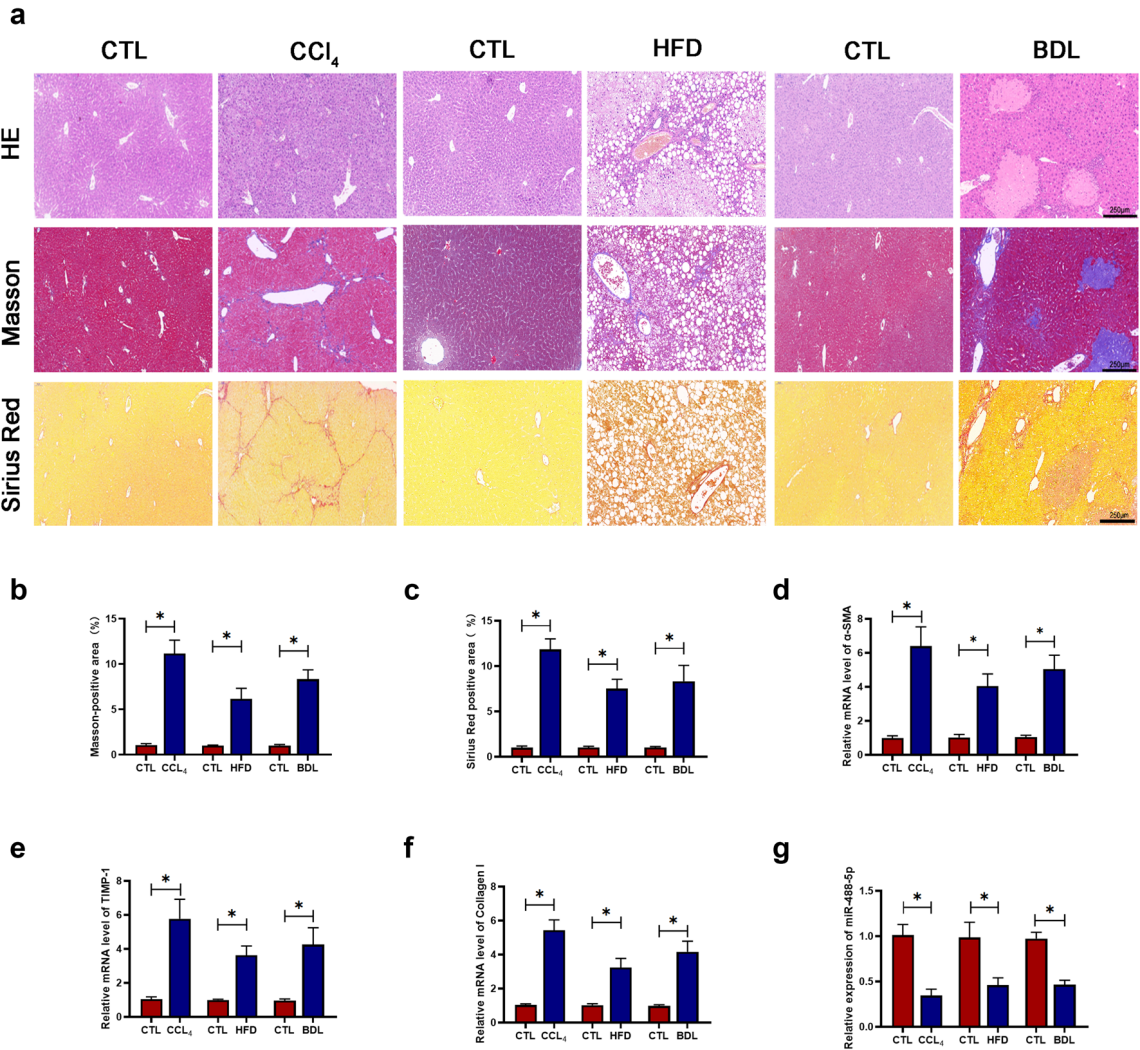
miR-488-5p is downregulated in multiple hepatic fibrosis models. Hepatic fibrosis models including CCl4, HFD and BDL were established for further research. **a** HE, Masson and Sirius Red staining of hepatic specimens from CCl4 (8 weeks), HFD (24 weeks) and BDL (2 weeks). **b**, **c** The Masson-positive and Sirius Red-positive areas were quantified in each group. **d**–**f** qRT-PCR analysis of the mRNA level of α-SMA, TIMP-1 and collagen-I in each group. **g** qRT-PCR analysis of the mRNA level of miR-488-5p in each group. Data represent means ± SEM of at least three independent experiments. *p value < 0.05

For purpose of detecting the effect of miR-488-5p in hepatic fibrotic tissues, we established three mouse liver fibrosis models. CCl_4_, BDL and HFD were administrated to the mice to, respectively, develop liver fibrosis. As shown in the Fig. 2a–c, HE, Sirius Red and Masson staining results presented the severity of fibrotic level caused by CCl_4_, BDL and HFD. Compared with the respective control (CTL) group, the histological structure was seriously damaged in CCl_4_ and BDL group, meanwhile the lipid vacuoles were abundant in the HFD group. At the same time the massive deposition of collagen was presented in CCl_4_, HFD and BDL group. Compared to their respective CTL group, the mRNA level of fibrosis-related markers containing α-SMA, tissue inhibitor of metalloproteinases 1 (TIMP-1) and Collagen-I were significantly upregulated (Fig. 2d–f). In the meantime, the level of miR-488-5p was decreased in the CCl_4_, BDL and HFD group, compared with their respective CTL group (Fig. 2g). To sum up, our study presented that miR-488-5p is downregulated in multiple liver fibrosis models, indicating that miR-488-5p participates in the development of hepatic fibrosis.

### miR-488-5p regulates cellular proliferation, apoptosis and activation level of HSCs

**Fig. 3:**
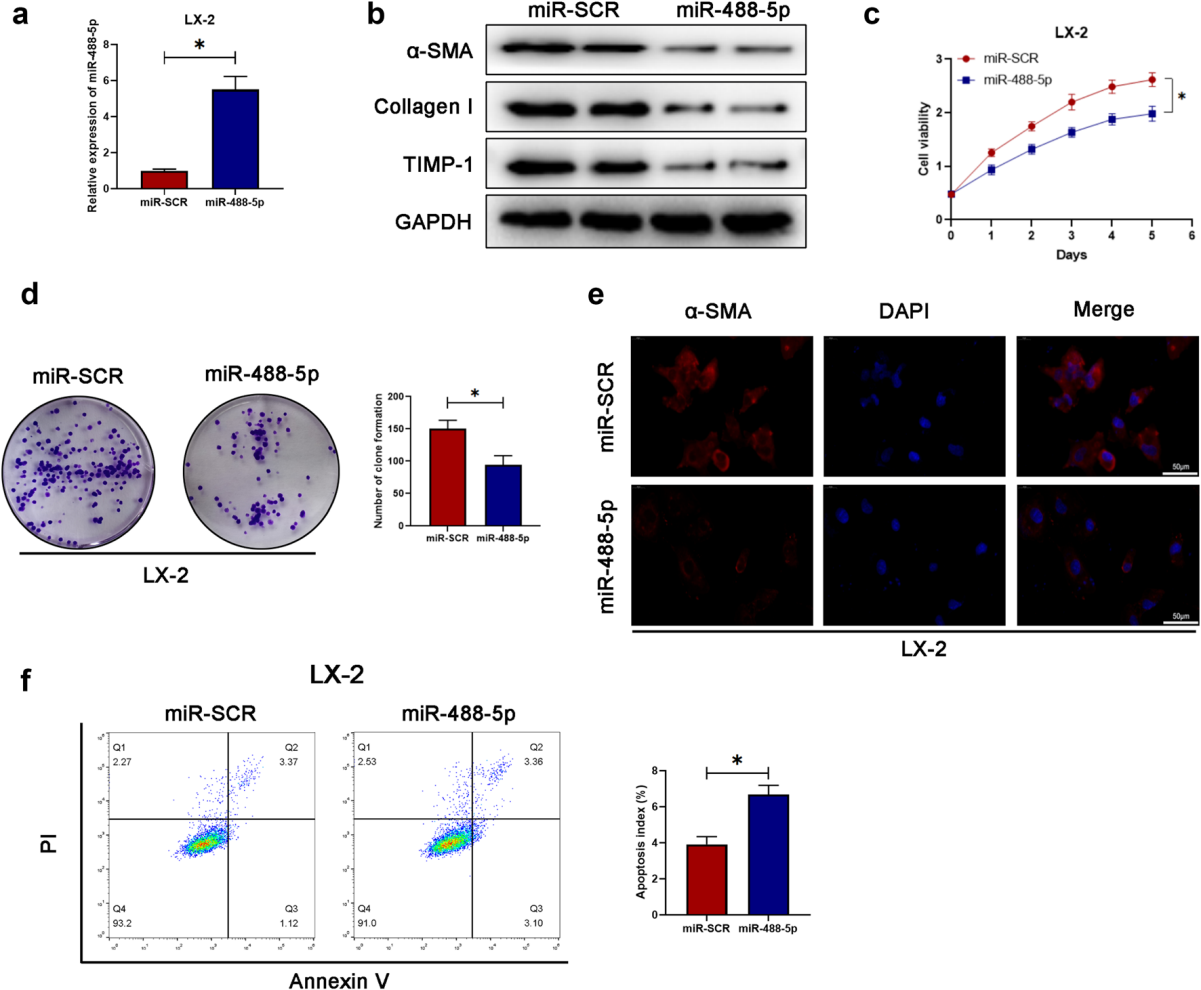
miR-488-5p regulates cellular proliferation, apoptosis and activation level of HSCs. **a** qRT-PCR analysis of the mRNA level of miR-488-5p in LX-2 cells. **b** WB analysis of the protein level of α-SMA, collagen-I and TIMP-1 in LX-2 cells. **c** Detection of the cell viability was dependent on CCK8 assay. **d** Clone formation of LX-2 cells and quantified. **e** IF tests the expression of α-SMA in LX-2 cells. **f** Flow cytometry evaluates the apoptosis index of LX-2 cells and the quantification. Data represent means ± SEM of at least three independent experiments. *p value < 0.05

For further determining the precise effect of miR-488-5p in the activated HSCs during fibrosis, we transfected the LX-2 cells with miR-SCR as control or miR-488-5p mimics. As presented in the Fig. 3a, the relative level of miR-488-5p was remarkably increased in LX-2 cells after miR-488-5p mimics transfection. As presented in the Fig. 3b, LX-2 cells with miR-488-5p overexpression were characterized with the lower protein level of fibrosis-related markers which contained a-SMA, TIMP-1, and collagen-I. Moreover, the cellular proliferative capability was inhibited in the LX-2 cells with miR-488-5p overexpression, based on the cell viability and clone formation results (Fig. 3c, d). At the same time, the IF staining revealed that the lower intensity of a-SMA was found in the LX-2 cells with miR-488-5p overexpression (Fig. 3e). Meanwhile, transfection with miR-488-5p mimics could result in the higher apoptosis index of LX-2 cells (Fig. 3f). As mentioned above, miR-488-5p overexpression was able to contribute to the repressed expression of fibrotic markers, cellular viability and the promoted apoptotic level of HSCs.

### TET3 is a direct target of miR-488-5p and upregulated in activated HSCs and fibrotic liver tissues

**Fig. 4:**
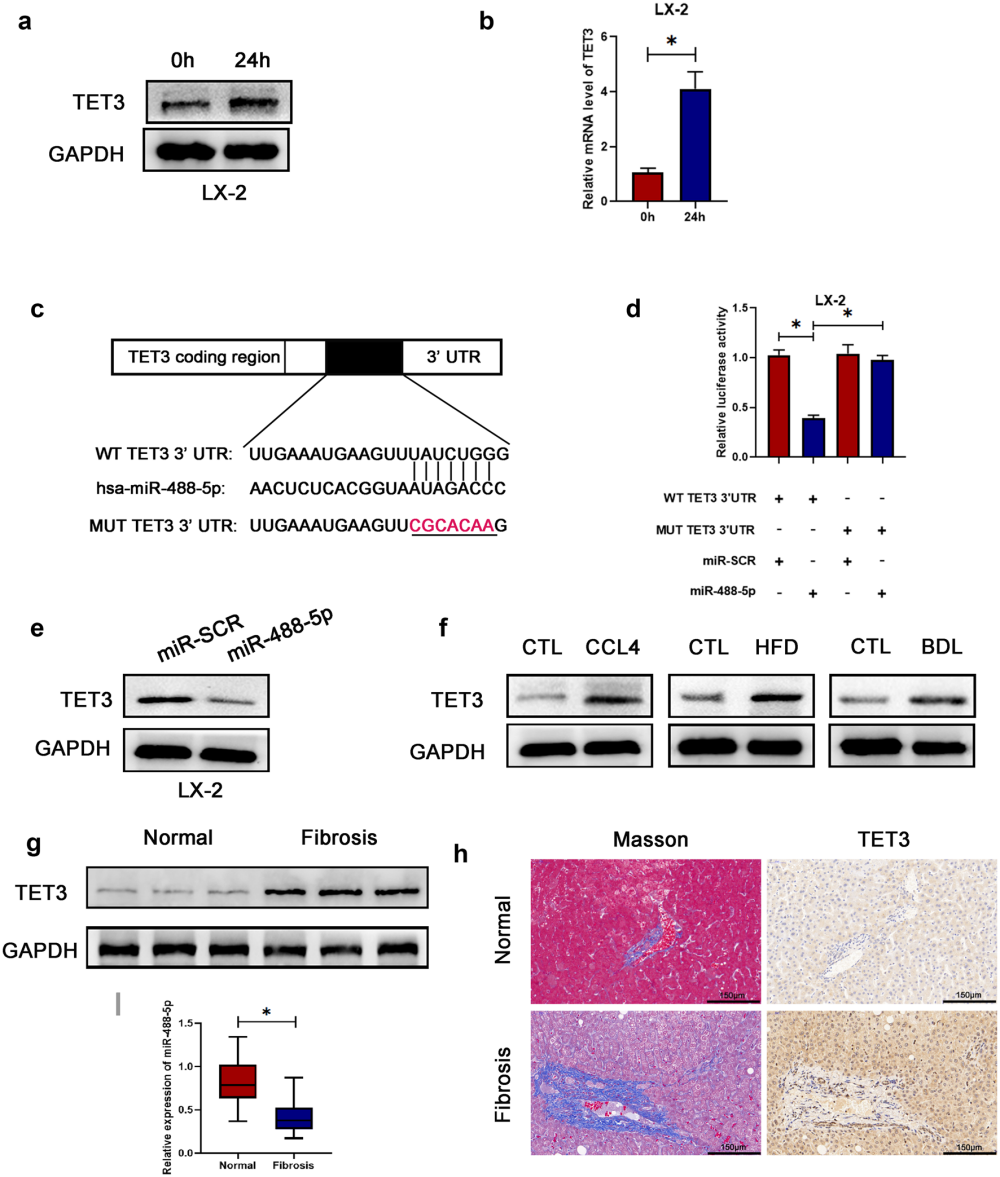
miR-488-5p targets TET3 via binding onto the 3′ UTR of TET3 mRNA directly. **a**, **b** WB and qRT-PCR analysis of the expression level of TET3 in LX-2 cells activated by TGF-β1 (10 ng/mL) for 0 and 24 h. **c** Predicted miR-488-5p targeting sequence in TET3 3′UTR (WT TET3 3′UTR). Target sequences of TET3 3′UTR were mutated (MUT TET3 3′UTR). **d** Dual-luciferase reporter assay of LX-2 cells transfected with WT TET 3′UTR or MUT TET3 3′UTR reporter together with miR-488-5p mimics or miR-SCR. **e** WB analysis of the expression of TET3 in LX-2 cells transfected with miR-488-5p mimics or miR-SCR. **f** WB analysis of the expression of TET3 in CCl4, HFD and BDL group as well as their respective control (CTL) group. **g** WB analysis of the expression of TET3 in normal and fibrotic liver tissues from patients. **h** Immunohistochemical staining of Masson and TET3 in hepatic samples from patients. **i** qRT-PCR analysis of the expression level of miR-488-5p in normal and fibrotic liver tissues from patients. Data represent means ± SEM of at least three independent experiments. *p value < 0.05

For the purpose of elucidating the underlying mechanism of miR-488-5p regulating HSCs activation, the online bioinformatics analyses for seeking the target gene of miRNA were performed. The intersection of two databases, containing miRWalk and TargetScan, showed that tet methylcytosine dioxygenase 3 (TET3) was a probable target gene of miR-488-5p. After consulting plentiful literatures, we found that tet methylcytosine dioxygenase 3 (TET3) was closely related with fibrosis [14, 15]. For the purpose of determining whether the effect of miR-488-5p was dependent on TET3, first, we performed the WB and qRT-PCR assays and our data revealed that the relative level of TET3 were increased in HSCs activated by TGF-β1 for 24 h (Fig. 4a, b). After performing the dual‐luciferase reporter assay, we observed that the co‐transfection with miR‐488-5p mimic and pGL3‐TET3‐WT 3′UTR contributed to the reduced luciferase activity (Fig. 4c, d). Meanwhile, the expression of TET3 was remarkably repressed in the miR‐488-5p-overexpressed LX-2 cells (Fig. 4e). Subsequently, the higher protein level of TET3 was detected in three hepatic fibrosis models (Fig. 4f). Moreover, the expression of TET3 was remarkably higher in patients with hepatic fibrosis, based on the WB, Masson and IHC staining (Fig. 4g, h). Meanwhile, the qRT-PCR data revealed that miR‐488-5p was downregulated in human fibrotic liver tissues which indicated miR-488-5p could serve as a potential target for hepatic fibrosis (Fig. 4i). Collectively, the data identified that TET3, as a direct target of miR‐488-5p, were remarkably upregulated in activated HSCs, liver fibrosis models and fibrotic liver specimens from the patients.

### Effect of miR-488-5p in HSCs activation depends on TET3

**Fig. 5:**
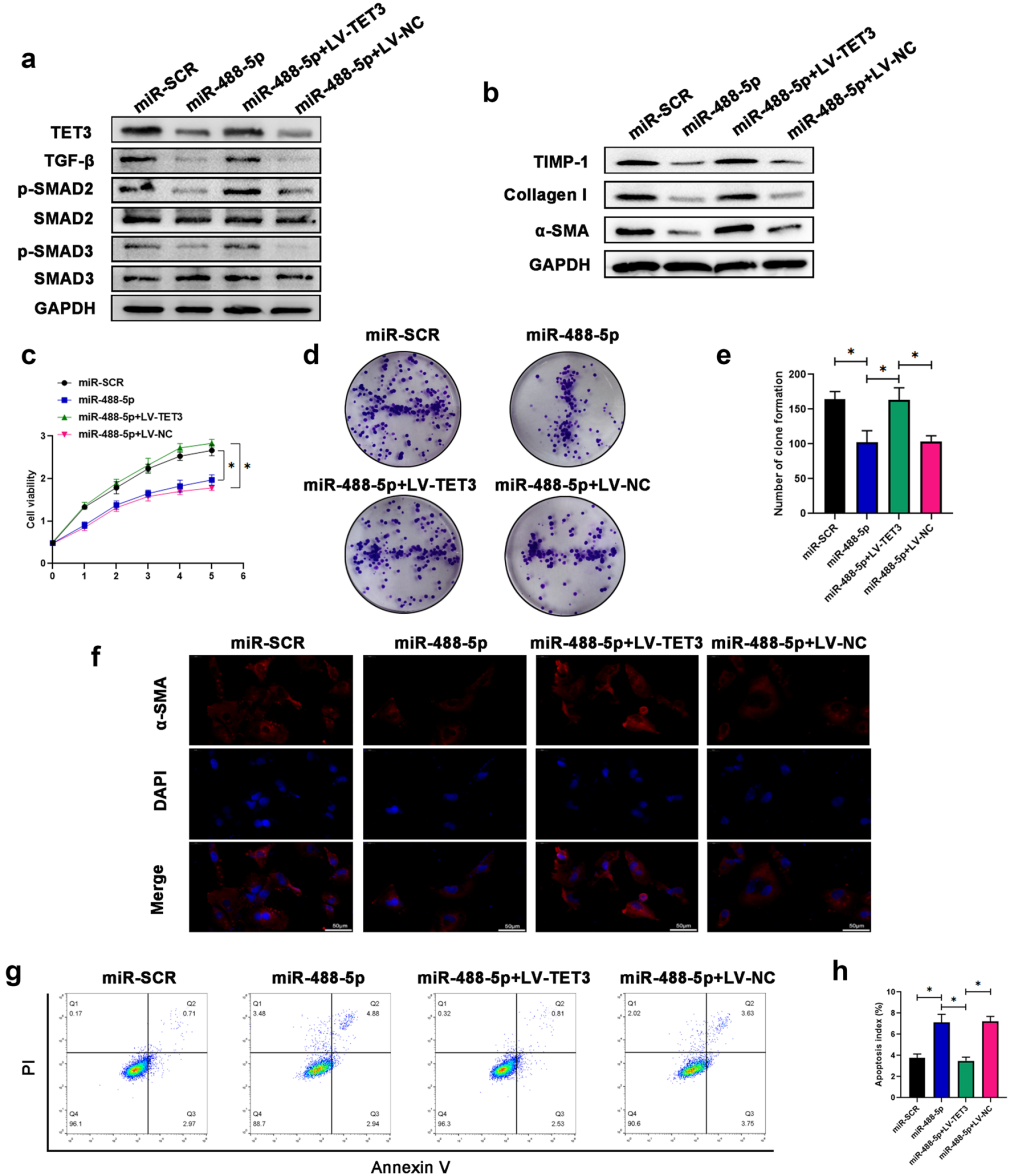
The effect of miR-488-5p in HSCs activation depends on TET3. LX-2 cells transfected with miR-SCR or miR-488-5p mimics or miR-488-5p mimics + LV-TET3 or miR-488-5p mimics + LV-NC were activated by TGF-β1 (10 ng/mL) for 24 h for further research. **a** WB analysis of the relative expression of TET3, TGF-β, p-SMAD2, SMAD2, p-SMAD3 SMAD3 in LX-2 cells from each group. **b** WB analysis of the relative protein level of TIMP-1, collagen-I and α-SMA in LX-2 cells from each group. **c** The cell viability of LX-2 cells from each group was detected by CCK8 assay. **d**, **e** Clone formation of LX-2 cells was quantified in each group. **f** IF analysis of the expression of α-SMA in LX-2 cells from each group. **g**, **h** Flow cytometry evaluates the apoptosis index of LX-2 cells and the quantification. Data represent means ± SEM of at least three independent experiments. *p value < 0.05

Few studies have reported that TET3 played an essential role in activating TGF-β pathway, which is closely involved in the liver fibrosis [14, 16]. For the purpose of further judging that the effect of miR-488-5p in HSCs activation depended on TET3, LV-TET3 or LV-NC was used to transfect the miR‐488-5p-overexpressed LX-2 cells. As shown in the Fig. 5a, as the expression of TET3 decreased in the miR‐488-5p-overexpressed LX-2 cells, so did the expression of TGF-β, p-SMAD2 and p-SMAD3. Meanwhile, overexpression of TET3 in LX-2 could contribute to reversing the inhibitory effect of miR-488-5p in TGF-β/SMAD2/3 pathway. In addition, the expression of fibrosis-related makers containing TIMP-1, collagen-I and a-SMA was upregulated in the LX-2 cells transfected with miR-488-5p mimics and LV-TET3 (Fig. 5b). Then, we performed the CCK8 assay and clone formation assay; the data showed that the cellular proliferative capabilities were promoted in the LX-2 cells transfected with miR-488-5p mimics and LV-TET3 (Fig. 5c–e). At the same time, the IF staining data revealed that the higher intensity of a-SMA was detected in the LX-2 cells transfected with miR-488-5p mimics and LV-TET3 (Fig. 5f). Meanwhile, the promoted apoptosis index of the miR‐488-5p-overexpressed LX-2 cells was counteracted by TET3 overexpression (Fig. 5g, h). Collectively, our data demonstrated that the effect of miR-488-5p in HSCs activation depended on TET3.

### miR-488-5p contributes to the reduced degree of hepatic fibrosis

**Fig. 6:**
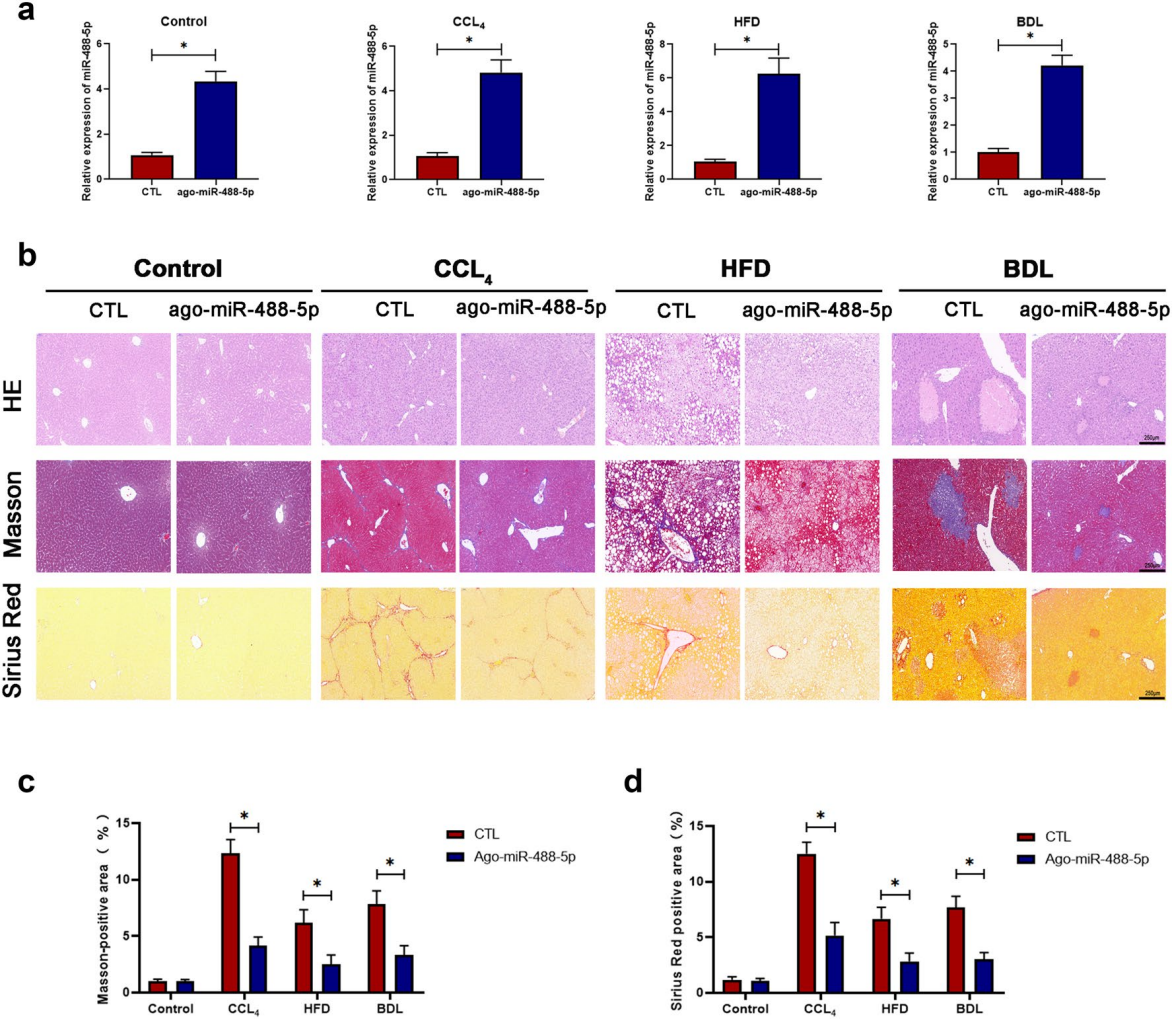
miR-488-5p contributes to the reduced degree of liver fibrosis. The mice in the control, CCl4, BDL and HFD groups were treated with ago-miR control (CTL) or ago-miR-488-5p. **a** The mRNA level of miR-488-5p in each group was detected in by qRT-PCR. **b** HE, Masson and Sirius Red staining of liver sections from the mice in each group. **c**, **d** The quantification of Masson-positive and Sirius Red-positive areas in each group. Data represent means ± SEM of at least three independent experiments. *p value < 0.05

Based on our results described above, we discovered that miR-488-5p participated in the HSCs proliferation, apoptosis and activation in vitro. For the purpose of sequentially exploring the effect of miR-488-5p in liver fibrosis in vivo, the mice from Control, CCl_4_, HFD and BDL group were, respectively, subjected to the transfection with ago-miR control (CTL) and ago-miR-488-5p. As presented in the Fig. 6a, the qRT-PCR results revealed that the level of miR-488-5p was upregulated in the group treated with ago-miR-488-5p. Meanwhile, our data revealed that miR-488-5p overexpression was helpful to mitigate liver fibrosis, as the histological structural damage was significantly alleviative in CCl_4_ and BDL group; meanwhile, the number of lipid vacuoles was reduced in the HFD group (Fig. 6b). Moreover, the reduced amount of collagen deposition was demonstrated in the liver tissues from CCl_4_, HFD and BDL group which were transfected with ago-miR-488-5p, according to the Masson and Sirius Red staining results (Fig. 6b–d). Therefore, the above data suggested that miR-488-5p alleviated liver fibrosis from multiple liver fibrosis models.

### miR-488-5p inhibits TET3 expression in fibrotic liver tissues

**Fig. 7:**
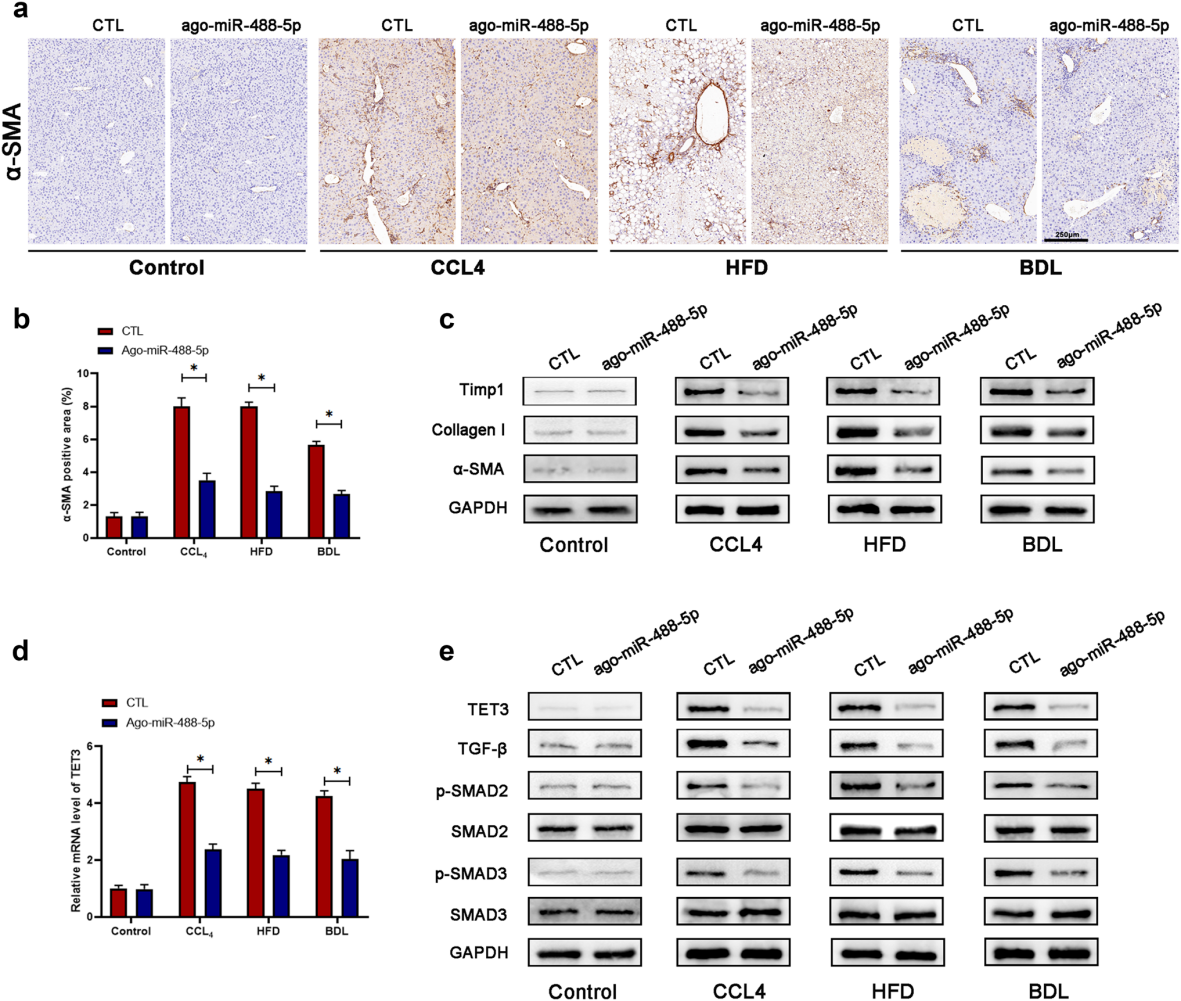
miR-488-5p inhibits the expression of TET3 in liver fibrosis tissues. **a**, **b** The expression of α-SMA in liver sections from the mice in each group was detected by immunohistochemical staining and quantified. **c** WB analysis of the relative expression of TIMP-1, collagen-I and α-SMA in liver tissues of mice in each group. **d** qRT-PCR analysis of the level of TET3 in liver tissues of mice in each group. **e** WB analysis of the expression of TET3, TGF-β, p-SMAD2, SMAD2, p-SMAD3 SMAD3 in liver tissues of mice in each group. Data represent means ± SEM of at least three independent experiments. *p value < 0.05

To identify that miR-488-5p inhibited liver fibrosis by targeting TET3 which was related with TGF-β/SMAD2/3 pathway, first, we evaluated the α-SMA expression of liver tissues through IHC staining; our data showed that α-SMA expression was remarkably inhibited in the liver tissues from CCl_4_, HFD and BDL group which were transfected with ago-miR-488-5p (Fig. 7a, b). Next, the protein expression of Timp1, collagen I and α-SMA was detected by WB assay which presented that miR-488-5p overexpression could significantly inhibit the expression of the above fibrosis-related makers in liver fibrosis tissues (Fig. 7c). Sequentially, the qRT-PCR analysis revealed that relative mRNA level of TET3 in liver tissues was significantly lower in the CCl_4_, HFD and BDL group which were transfected with ago-miR-488-5p compared to those transfected with ago-miR control (CTL) (Fig. 7d). Eventually, the WB results showed transfection with ago-miR-488-5p could reduce the expression of TET3, TGF-β, p-SMAD2 and p-SMAD3 in the fibrotic liver samples from CCl_4_, HFD and BDL group. To sum up, our study determined that miR-488-5p mitigates liver fibrosis by inhibiting TET3/TGF-β/SMAD2/3 pathway.

## Discussion

Hepatic fibrosis, as a common pathological condition in clinic, is usually caused by the long-term exposure to viral hepatitis, excessive drinking, fat-rich diet, drug-induced liver injury and biliary obstruction, and so on [17–19]. One of the most typical features of hepatic fibrosis is the existence of massive ECM deposition largely caused by the activated HSCs [20, 21].

The role of miRNAs in HSCs activation and hepatic fibrosis has attracted plenty of attention for the past few years [22, 23]. For instance, miR-34a promotes HSCs activation and hepatic fibrosis via the TGF-β pathway [24]. Upregulation of miR-125b reduced endoplasmic reticulum stress induced by obstructive jaundice-induced liver fibrosis [25]. It was reported that miR-494-3p inhibits HSCs activation and hepatic fibrosis via targeting TRAF3 which resulted in the impaired proliferation and enhanced apoptosis [26]. Moreover, the inhibition of methyltransferase caused by miR-29a overexpression could prevent hepatic fibrosis induced by BDL [27]. Even so, the role of miRNAs in hepatic fibrosis is still indistinct and needs to be concerned.

Through referring many literatures concerning miRNA and liver fibrosis, we noticed that the microarray analysis for miRNA expression from a previous study revealed miR-488-5p was significantly downregulated in activated LX-2 cells which hinted that miR-488-5p may be involved with the progression of liver fibrosis [28]. Our data identified that downregulation of miR-488-5p in LX-2 cells activated by TGF-β1 was detected. And miR-488-5p overexpression could contribute to the repressed proliferative capacity and enhanced apoptosis index of LX-2 cells. Moreover, the data demonstrated that miR-488-5p was also remarkably downregulated in CCl_4_, BDL and HFD models and human fibrotic liver samples. Therefore, our study indicated that miR-488-5p might play a vital role in inhibiting hepatic fibrosis, which was worthy of further study.

Bioinformatics analyses were executed to explore the underlying target of miR-488-5p through miRWalk and TargetScan, the further experiments indicated that tet methylcytosine dioxygenase 3 (TET3) was the direct target of miR-488-5p in LX-2 cells. TET3 belongs to the member of ten–eleven translocation (TET) family proteins that participate in oxidizing 5-methylcytosine (5mC) into 5-hydroxymethylcytosine (5hmC) [29]. It had been recently reported that TET3 was tightly related to the process of epigenetic modulation, embryonic development, stem cell renewal, and tumor [30–34]. Meanwhile, some studies have put attention on the role of TET3 in fibrosis, especially hepatic fibrosis. As reported before, the TET3/TGF-β loop enhanced HSCs activation and ECM production [14]. Based on our data, TET3 was verified to be the target of miR-488-5p which bound onto the 3’ UTR of TET3 mRNA directly. Our data revealed that miR-488-5p overexpression repressed TET3 expression in the activated LX-2 cells. Moreover, the upregulation of TET3 was detected in multiple hepatic fibrosis models and human fibrotic liver tissues.

As we know, TGF-β participates in the plenty of biological processes, containing cellular migration, development, proliferation and differentiation, and so on [35]. Moreover, the effect of TGF-β, as the key activator of HSCs activation, has been extensively reported in the development of hepatic fibrosis for decades [36, 37]. Numerous studies have revealed that TGF-β/SMAD2/3 signaling pathway is closely related to HSCs activation and fibrogenesis, for example, piperine inhibited HSCs activation and mitigated hepatic fibrosis via activating Nrf2 which sequentially repressed TGF-β/SMAD2/3 signaling pathway [38]. Also, LOX-like 1 enhanced HSCs activation through TGF-β-induced proliferation and fibrogenesis by promotion of SMAD2/3 phosphorylation [39]. Our data demonstrated that the inhibitory effect of miR-488-5p in HSCs activation in vitro through restraining TET3/TGF-β/SMAD2/3 pathway, also TET3 overexpression abolished the protective role of miR-488-5p which inhibited proliferative capability and enhanced apoptosis index. At the same time, miR-488-5p could mitigate liver fibrosis in vivo through inhibiting TGF-β/SMAD2/3 pathway.

In conclusion, we identified that miR-488-5p, as a fibrosis-inhibitory factor, was downregulated in the activated HSCs and fibrotic liver tissues. miR-488-5p overexpression restrained HSCs activation and hepatic fibrosis via restraining TET3/TGF-β/SMAD2/3 signaling pathway.

## Supplementary Information

Below is the link to the electronic supplementary material.

Supplementary file1 (DOCX 16 KB)

## Data Availability

All data that obtained and analyzed during our study are available from the corresponding author once reasonably requested.

## References

[1] Benyon RC, Iredale JP (2000). Is liver fibrosis reversible?. Gut.

[2] Ginès P (2022). Population screening for liver fibrosis: toward early diagnosis and intervention for chronic liver diseases. Hepatology.

[3] Yi J (2021). Berberine alleviates liver fibrosis through inducing ferrous redox to activate ROS-mediated hepatic stellate cells ferroptosis. Cell Death Discov.

[4] Shi Z (2020). Transcriptional factor ATF3 promotes liver fibrosis via activating hepatic stellate cells. Cell Death Dis.

[5] Yang A (2021). Hepatic stellate cells-specific LOXL1 deficiency abrogates hepatic inflammation, fibrosis, and corrects lipid metabolic abnormalities in non-obese NASH mice. Hepatol Int.

[6] Wang X (2020). Roseotoxin B alleviates cholestatic liver fibrosis through inhibiting PDGF-B/PDGFR-β pathway in hepatic stellate cells. Cell Death Dis.

[7] Zhang J (2021). Sirt6 alleviated liver fibrosis by deacetylating conserved lysine 54 on Smad2 in hepatic stellate cells. Hepatology.

[8] Xi S (2021). Activated hepatic stellate cells induce infiltration and formation of CD163(+) macrophages via CCL2/CCR2 pathway. Front Med (Lausanne).

[9] Preethi KA (2022). Liquid biopsy: exosomal microRNAs as novel diagnostic and prognostic biomarkers in cancer. Mol Cancer.

[10] Zhang D (2021). CircRNA-vgll3 promotes osteogenic differentiation of adipose-derived mesenchymal stem cells via modulating miRNA-dependent integrin α5 expression. Cell Death Differ.

[11] Wang H (2021). miRNA-130b-5p promotes hepatic stellate cell activation and the development of liver fibrosis by suppressing SIRT4 expression. J Cell Mol Med.

[12] Ma Y (2022). miR-98–5p as a novel biomarker suppress liver fibrosis by targeting TGFβ receptor 1. Hepatol Int.

[13] Yu K (2018). miR-96-5p prevents hepatic stellate cell activation by inhibiting autophagy via ATG7. J Mol Med (Berl).

[14] Xu Y (2020). A positive feedback loop of TET3 and TGF-β1 promotes liver fibrosis. Cell Rep.

[15] Zhang QQ (2014). TET3 mediates the activation of human hepatic stellate cells via modulating the expression of long non-coding RNA HIF1A-AS1. Int J Clin Exp Pathol.

[16] Yang L (2020). Activation of BK channels prevents hepatic stellate cell activation and liver fibrosis through the suppression of TGFβ1/SMAD3 and JAK/STAT3 profibrotic signaling pathways. Front Pharmacol.

[17] Hernandez-Gea V, Friedman SL (2011). Pathogenesis of liver fibrosis. Annu Rev Pathol.

[18] Karl M (2022). Pathogenesis of liver fibrosis. Hepatology.

[19] Liu Z (2022). Diagnosis of significant liver fibrosis in patients with chronic hepatitis B using a deep learning-based data integration network. Hepatol Int.

[20] Bataller R, Brenner DA (2005). Liver fibrosis. J Clin Investig.

[21] Hung CT (2022). Targeting ER protein TXNDC5 in hepatic stellate cell mitigates liver fibrosis by repressing non-canonical TGFβ signalling. Gut.

[22] Tai Y (2021). Integrated analysis of hepatic miRNA and mRNA expression profiles in the spontaneous reversal process of liver fibrosis. Front Genet.

[23] Wang X (2021). MicroRNAs as regulators, biomarkers and therapeutic targets in liver diseases. Gut.

[24] Zhang J (2021). MiR-34a promotes fibrosis of hepatic stellate cells via the TGF-β pathway. Ann Transl Med.

[25] Zhang X (2021). miRNA-125b signaling ameliorates liver injury against obstructive jaundice-induced excessive fibrosis in experimental rats. Yonsei Med J.

[26] Li H (2021). MicroRNA-494-3p prevents liver fibrosis and attenuates hepatic stellate cell activation by inhibiting proliferation and inducing apoptosis through targeting TRAF3. Ann Hepatol.

[27] Yang YL (2017). MicroRNA-29a alleviates bile duct ligation exacerbation of hepatic fibrosis in mice through epigenetic control of methyltransferases. Int J Mol Sci.

[28] Wei S (2019). miR-455-3p alleviates hepatic stellate cell activation and liver fibrosis by suppressing HSF1 expression. Mol Ther Nucleic Acids.

[29] Antunes C (2021). Tet3 ablation in adult brain neurons increases anxiety-like behavior and regulates cognitive function in mice. Mol Psychiatry.

[30] He YF (2011). Tet-mediated formation of 5-carboxylcytosine and its excision by TDG in mammalian DNA. Science.

[31] Tan L, Shi YG (2012). Tet family proteins and 5-hydroxymethylcytosine in development and disease. Development.

[32] Cimmino L (2011). TET family proteins and their role in stem cell differentiation and transformation. Cell Stem Cell.

[33] Pulikkottil AJ (2022). TET3 promotes AML growth and epigenetically regulates glucose metabolism and leukemic stem cell associated pathways. Leukemia.

[34] Liu Y (2021). The KRAS/Lin28B axis maintains stemness of pancreatic cancer cells via the let-7i/TET3 pathway. Mol Oncol.

[35] Morikawa M, Derynck R, Miyazono K (2016). TGF-β and the TGF-β family: context-dependent roles in cell and tissue physiology. Cold Spring Harb Perspect Biol.

[36] Kyritsi K (2021). Mast cells induce Ductular reaction mimicking liver injury in mice through mast cell-derived transforming growth factor beta 1 signaling. Hepatology.

[37] Yang X (2020). Twist1-induced miR-199a-3p promotes liver fibrosis by suppressing caveolin-2 and activating TGF-β pathway. Signal Transduct Target Ther.

[38] Shu G (2021). Piperine inhibits AML-12 hepatocyte EMT and LX-2 HSC activation and alleviates mouse liver fibrosis provoked by CCl(4): roles in the activation of the Nrf2 cascade and subsequent suppression of the TGF-β1/Smad axis. Food Funct.

[39] Ma L (2018). Knockdown of LOXL1 inhibits TGF-β1-induced proliferation and fibrogenesis of hepatic stellate cells by inhibition of Smad2/3 phosphorylation. Biomed Pharmacother.

