# Supplementary material for: miR-488-5p mitigates hepatic stellate cell activation and hepatic fibrosis via suppressing TET3 expression
Source: Hepatol Int. 2022 Aug 24;17(2):463–75. doi: 10.1007/s12072-022-10404-w (PMC10119239; doi:10.1007/s12072-022-10404-w)
Supplement: Supplementary file 1 — Supplementary file1 (DOCX 16 KB) [file 12072_2022_10404_MOESM1_ESM.docx]

**Supplementary table 1: Primer sequences for the amplification**.

| Gene | Forward Primer（5′ → 3′） | Reverse Primer（5′ → 3′） |
| --- | --- | --- |
| miR-488-5p  (Human) | CCCAGATAATGGCACTC | GAACATGTCTGCGTATCTC |
| U6(Human) | ATTGGAACGATACAGAGAAGATT | GGAACGCTTCACGAATTTG |
| TET3(Human) | GACGAGAACATCGGCGGCGT | GTGGCAGCGGTTGGGCTTCT |
| β-actin  (Human) | CATGTACGTTGCTATCCAGGC | CTCCTTAATGTCACGCACGAT |
| miR-488-5p  (Mouse) | ACACTCCAGCTGGGTTGAAAGGCTGTTTC | TGGTGTCGTGGAGTCG |
| U6(Mouse) | GCTTCGGCACATATACTAAAAT | CGCTTCACGAATTTGCGTGTCAT |
| TET3(Mouse) | TGCGATTGTGTCGAACAAATAGT | TCCATACCGATCCTCCATGAG |
| α-SMA  (Mouse) | GTCCCAGACATCAGGGAGTAA | TCGGATACTTCAGCGTCAGGA |
| Collagen-I  (Mouse) | GCTCCTCTTAGGGGCCACT | CCACGTCTCACCATTGGGG |
| TIMP-1  (Mouse) | GCAACTCGGACCTGGTCATAA | CGGCCCGTGATGAGAAACT |
| β-actin  (Mouse) | GGCTGTATTCCCCTCCATCG | CCAGTTGGTAACAATGCCATGT |
